# Imaging fast electrical activity in the brain during ictal epileptiform discharges with electrical impedance tomography

**DOI:** 10.1016/j.nicl.2018.09.004

**Published:** 2018-09-05

**Authors:** Sana Hannan, Mayo Faulkner, Kirill Aristovich, James Avery, Matthew Walker, David Holder

**Affiliations:** a Department of Medical Physics and Biomedical Engineering, University College London, UK; b Institute of Neurology, University College London, UK

**Keywords:** Electrical impedance tomography, Epilepsy, Spike-and-wave discharge, Cerebral cortex, Rat

## Abstract

Electrical Impedance Tomography (EIT) is an emerging medical imaging technique which can produce tomographic images of internal impedance changes within an object using non-penetrating surface electrodes. It has previously been used to image impedance changes due to neuronal depolarisation during evoked potentials in the rat somatosensory cortex with a resolution of 2 ms and <200 μm, using an epicortical electrode array. The purpose of this work was to use this technique to elucidate the intracortical spatiotemporal trajectory of ictal spike-and-wave discharges (SWDs), induced by electrical stimulation in an acute rat model of epilepsy, throughout the cerebral cortex. Seizures lasting 16.5 ± 5.3 s with repetitive 2–5 Hz SWDs were induced in five rats anaesthetised with fentanyl-isoflurane. Transfer impedance measurements were obtained during each seizure with a 57-electrode epicortical array by applying 50 μA current at 1.7 kHz to two electrodes and recording voltages from all remaining electrodes. Images were reconstructed from averaged SWD-related impedance traces obtained from EIT measurements in successive seizures. We report the occurrence of reproducible impedance changes during the initial spike phase, which had an early onset in the whisker barrel cortex and spread posteriorly, laterally and ventrally over 20 ms (*p* < 0.03125, *N* = 5). These findings, which confirm and extend knowledge of SWD initiation and expression, suggest that EIT is a valuable neuroimaging tool for improving understanding of neural circuits implicated in epileptic phenomena.

## Highlights

• Ictal spike-and-wave discharges were imaged in the rat brain using EIT.
• The propagation pattern of activity through the cerebral cortex was characterised.
• Activity propagated posterolaterally and ventrally from the whisker barrel cortex.
• Mechanisms of other epileptiform activities may also be elucidated with these methods.

## 1 Introduction

### 1.1 Background

#### 1.1.1 Intracranial ECoG and scalp EEG for intractable epilepsy

20–40% of individuals with epilepsy are resistive to anticonvulsant medication for complete seizure control and a proportion of these may benefit from neurosurgical resection of epileptogenic tissue (Nowell et al., 2014; Nair, 2016). Prior to resective surgery, high-resolution MRI imaging to detect structural abnormalities and long-term video-EEG telemetry may be utilised to localise the ictal onset zone in individuals with intractable focal epilepsies (Ryvlin and Rheims, 2008). However, in many cases, such as non-lesional or extratemporal lobe epilepsy, scalp EEG recording is not adequate for localising epileptogenic foci and additional invasive telemetry methods are necessary (Duncan, 2011). This may be accomplished with implantation of subdural electrode mats on the exposed surface of the brain (electrocorticography (ECoG)) or insertion of depth electrodes into a lesion or the brain parenchyma (stereoencephalography) (Podkorytova et al., 2016). Distinctive ictal and/or interictal discharges, such as spikes, sharp waves and spike-and-wave discharges (SWDs), may be evident in these recordings (Nair, 2016). The SWD is a rhythmic and abnormal transient oscillatory event which comprises a spike component and an ensuing slow wave typically observed during seizures (Blumenfeld, 2005). SWDs are seen in many focal and generalized epilepsies, and are particularly common in typical childhood absence seizures, where they occur bilaterally at a frequency of 3–4 Hz (Panayiotopoulos, 2008). Of those individuals who undergo resective epilepsy surgery, under half remain seizure free ten years after surgery (de Tisi et al., 2011); postoperative seizure relapse can often be attributed to inaccuracies in localising foci (Spencer et al., 2005). Limitations of ECoG include the fact that epileptic discharges may not have electroencephalographic correlates if they originate from sources that are: located in deeper subcortical structures, oriented tangentially to the scalp, or extend over sulci or gyri with opposing source orientations, leading to selective cancellation of signals (Lüders, 2008; Schomer and Lopes da Silva, 2010; Ahlfors et al., 2011). Intracerebral depth electrodes, which can overcome these issues, are limited in their spatial sampling volume and, depending on their location, may cause functional deficits due to structural brain damage (Wellmer et al., 2012). There exists, therefore, an acute clinical need for improved presurgical evaluation to enable more precise localisation of the ictal onset zone.

#### 1.1.2 Initiation and propagation of spike-and-wave discharges in the cerebral cortex

While interictal spikes often define the irritative zone during presurgical evalulation for intractable epilepsy, this may be remote from the seizure onset zone itself which is better localised using ictal discharges, such as the SWD (Tyvaert et al., 2008). The spike component of SWDs is caused by an intense period of synchronised neuronal firing which produces a surface negative potential lasting up to 70 ms. The resulting hyperpolarisation phase produces the secondary slow wave, lasting around 100 ms and also surface negative, in which neural circuits are relatively quiescent (Blumenfeld, 2005). The origin of SWDs has been the source of much controversy, which may be attributed to differences between experimental models used. Earlier studies demonstrated a critical role for the highly interconnected circuitry of the cerebral cortex and thalamus and attributed the origin of these paroxysmal discharges to functional disturbances in these thalamocortical networks (Buzsáki, 1991; Avanzini et al., 2000; Blumenfeld, 2005). However, there is now a growing body of evidence suggesting a leading role of the cerebral cortex in the initiation and expression of SWDs (Steriade and Contreras, 1998; Timofeev et al., 1998; Meeren et al., 2002; Nersesyan et al., 2004; Sitnikova and van Luijetelaar, 2004; Timofeev and Steriade, 2004; Polack et al., 2007). More specifically, recent investigations in two genetic models of absence epilepsy, the WAG/Rij rat and Genetic Absence Epilepsy Rat from Strasbourg (GAERS), have shown that SWDs originate from a discrete focus within the facial region of the primary somatosensory cortex (Meeren et al., 2002; Meeren et al., 2005; Polack et al., 2007; Polack et al., 2009). *In vivo* intracellular recordings in GAERS revealed that pyramidal neurons in Layers V and VI of the facial somatosensory cortex are heavily implicated in generating spike-wave activity during absence seizures (Polack et al., 2007; Polack et al., 2009). However, the exact spatiotemporal trajectory of these oscillations through local and distant intracortical projections remains to be determined; such investigations have been hampered by the lack of a methodology capable of recording neuronal depolarisation over milliseconds and millimetres.

#### 1.1.3 EIT of fast electrical activity during neuronal depolarisation

Electrical Impedance Tomography (EIT) is an emerging medical imaging modality in which images of the internal electrical impedance of a subject can be reconstructed from multiple transfer impedance measurements made with non-penetrating surface electrodes (Holder, 2005). Each individual measurement is made by injecting current through a single electrode pair and recording the resulting boundary voltages from all remaining electrodes. EIT has the unique potential to image impedance changes which arise during fast electrical activity due to neuronal depolarisation. As neurons are activated, the opening of voltage- and ligand-gated ion channels in neuronal cell membranes enables current to pass into the intracellular space, manifesting as voltage changes on the recording electrodes. This technique has been used in the anaesthetised rat to produce impedance images of cortical neural activity during evoked somatosensory activity with a spatial and temporal resolution of 2 ms and <200 μm, using an epicortical electrode array (Aristovich et al., 2016). This method is limited by its depth penetration of 2 mm which means that sensitivity to impedance changes is generally restricted to the cerebral cortex, although simulations suggest that it may be possible to image fast electrical activity throughout the entire rat brain, with a localisation accuracy of <1 mm, using optimised experimental protocols (Aristovich et al., 2016).

EIT has been proposed, as an adjunct to conventional invasive or non-invasive EEG monitoring methods, for improving the preoperative localisation of epileptogenic foci in patients with medically refractory epilepsy who are candidates for surgery (Boone et al., 1994; Rao, 2000; Fabrizi et al., 2006). The fast impedance changes associated with individual epileptic discharges may be used for this purpose. Hypersynchronous depolarisation of local neuronal populations in the brain during these discharges is caused by the opening of voltage-dependent sodium and calcium ion channels (Stafstrom, 2007; Cain and Snutch, 2012). This enables current to pass across the cell membrane into activated neurons and, as a result, the recorded tissue impedance undergoes a rapid decrease lasting several milliseconds. This phenomenon was reported during interictal spikes induced by chemical models of epilepsy in the anaesthetised rat (Vongerichten et al., 2016). However, the cortical tissue impedance responses to ictal discharges have never been characterised or imaged.

Another impedance signal that can aid in localising the epileptogenic zone is the well-established longer-lasting change in cerebral tissue impedance over seconds during seizures (Van Harreveld and Schadé, 1962; Elazar et al., 1966; Rao, 2000; Olsson et al., 2006; Vongerichten et al., 2016; Wang et al., 2017; Hannan et al., 2018). This is caused by cell swelling, which can either precede the electrographic changes associated with epileptic discharges or occur shortly after the onset of such events (Andrew and MacVicar, 1994; Broberg et al., 2008; Binder and Haut, 2013). This longer-lasting impedance change is beyond the scope of the present study and so will not be addressed.

### 1.2 Purpose

To date, fast neural EIT has been used to produce images and describe the spatiotemporal propagation of neuronal activation during somatosensory evoked potentials in the cerebral cortex of the anaesthetised rat (Aristovich et al., 2016). The purpose of this work was to utilise a similar experimental approach to describe the trajectory of intracortical impedance changes during ictal spike-and-wave activity. Questions to be addressed were:

(i) Can EIT be used to produce biophysically plausible images of this activity?
(ii) If so, how does the propagation pattern of activity compare to current understanding of the initiation and expression of ictal SWDs in the cerebral cortex?

### 1.3 Experimental design

EIT was performed on one hemisphere of the anaesthetised rat brain using an epicortical array. A novelty of the current work, in comparison to previous EIT studies, is that a 57-electrode array was utilised, allowing for enhanced coverage of the neocortex and more unique current injection pairs, thus improving accuracy of reconstructed images. The cortical electrical stimulation model of epilepsy, which involves electrically stimulating the sensorimotor cortex (Supplementary Information 1.3), was used to induce epileptiform events comprising a train of repeatable SWDs, which will henceforth be termed ‘seizures’. This model was chosen as it enables frequent induction of seizures on demand, allowing for complete control of the EIT protocol (Nelson et al., 2010). Contrary to chemical models of epilepsy, intermittent electrical stimulation of cortical tissue does not induce irreversible plastic changes to local neural circuits; thus, time-dependent changes in seizure presentation are minimised (McNamara, 1986; Scaravilli, 1997; Schubert et al., 2005; Scharfman, 2008). Use of this model therefore enabled development of a method for measuring the impedance changes associated with neuronal depolarisation during ictal epileptiform discharges, an avenue which had not yet been explored.

An EIT measurement was obtained during each induced seizure; since the impedance change due to neuronal depolarisation is ~0.1–0.3% (Aristovich et al., 2016; Vongerichten et al., 2016), averaging of SWD-related impedance changes within each seizure was necessary to achieve a satisfactory signal-to-noise ratio (SNR) for imaging. Previous studies and modelling have indicated that an optimal dataset could be acquired with ≥30 current applications pairs, which ensured thorough sampling of the cortical volume. Since the electrographic presentation of seizures remained consistent during experiments, an independent current injection pair could be used per seizure. Therefore, an EIT image of an averaged SWD was reconstructed in each animal from data pooled across ≥30 seizures.

## 2 Materials and methods

### 2.1 Animal preparation

All animal handling and experimental investigations undertaken in this study were ethically approved by the UK Home Office and performed in accordance with its regulations, as outlined in the Animals (Scientific Procedures) Act 1986. Five adult female Sprague-Dawley rats (300–450 g) were used in total. Anaesthesia was induced with 4% isoflurane in 2 Lmin^−1^ O_2_ and an endotracheal intubation was performed to enable mechanical ventilation with 2–3% isoflurane in a 30/70 mixture of oxygen/air using an SAV03 ventilator (Vetronic Services Ltd., Abbotskerswell, UK). Cannulation of the right femoral vessels was undertaken to allow for monitoring of intra-arterial blood pressure and intravenous access. Exhaled gases, respiratory rate, tidal volume, heart rate, invasive arterial blood pressure and SpO_2_ were monitored regularly using an anaesthetic monitor (Lightning; Vetronic Services Ltd., Abbotskerswell, UK) and core body temperature was maintained at 36.5 ± 5 °C using a homeothermic heating unit (Harvard Apparatus, Edenbridge, UK). Rats were then fixed in a stereotaxic frame (Narishige International Ltd., London, UK), the skin of the head shaved and the scalp incised. The insertion of the temporal muscle on each side was cauterised using a bipolar coagulation unit (Codman Malis CMC-II; Codman, Raynham, MA) and incised with a scalpel. The cerebral cortex was exposed through a craniotomy in one hemisphere using a veterinary bone drill (Ideal Micro-Drill; Harvard Apparatus, Edenbridge, UK). The paramedial edge of the craniotomy extended from 1 mm anterior to lambda to 5 mm posterior to bregma, with the lateral boundary at the junction of the zygomatic to the temporal bone, forming a trapezoidal opening. The dura was incised and the exposed cortex frequently irrigated with 0.9% sterile saline at 37 °C until electrode implantation.

A planar custom-designed 57-contact epicortical electrode array was gently placed onto the exposed cortical surface using a micromanipulator (SM-15; Narishige International Ltd., London, UK). The array was trapezoidal in shape and measured 15 × 9 mm at its furthest edges, providing coverage of ~90% of the neocortex of one cerebral hemisphere (Supplementary Fig. S1, Supplementary Information 1.1, 1.2). The 57 electrodes, 0.6 mm in diameter, were platinised to produce a contact impedance of ≤5 kΩ across the electrode-electrolyte interface. A silver-silver chloride reference electrode, 9 mm in diameter, was placed beneath the nuchal skin. Following electrode implantation, anaesthesia was maintained with continuously-infused intravenous fentanyl at 20 μg/kg/h (Eurovet Animal Health Ltd., Cambridge, UK) and ~0.5% isoflurane.

### 2.2 Induction of seizures by cortical electrical stimulation

**Fig. 1:**
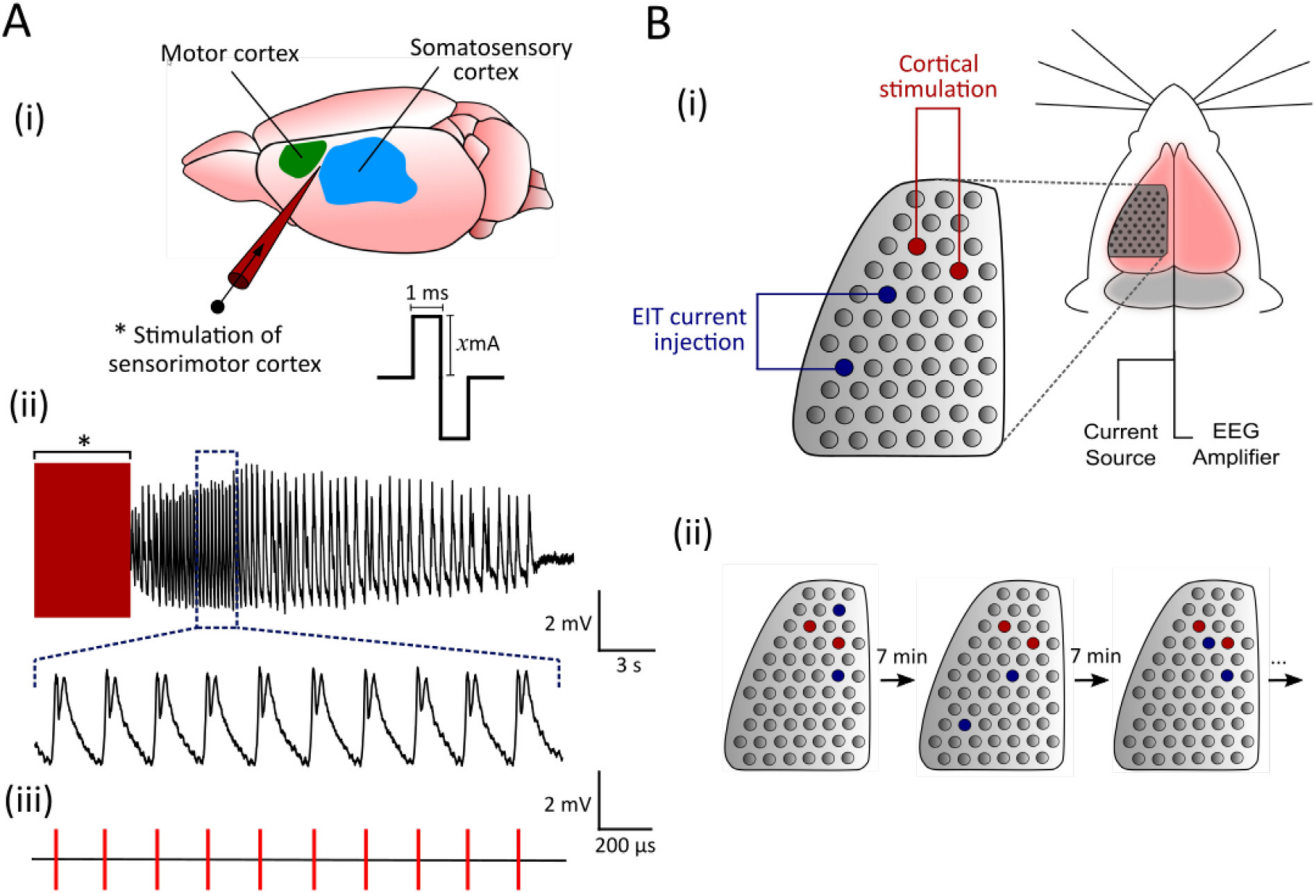
Schematic of experimental setup. A, The sensorimotor cortex was electrically stimulated with a 5-s train of 100 Hz biphasic square-wave pulses to induce seizures. Pulses were 1 ms in duration per phase and had current amplitude *x* (A(i)), defined as the threshold for ictal spike-and-wave ECoG activity. During post-acquisition signal processing, the ECoG trace recorded from the ictal focus (A(ii)) was used to set event markers at each peak of the spike component of ictal SWDs (A(iii)); these markers were used as triggers to construct averaged ECoG and impedance waveforms of a single SWD per seizure. B, A 57-electrode epicortical array was placed on the exposed cortical surface of an anaesthetised rat: two electrode pairs on the array were stimulated to induce seizures and obtain EIT measurements simultaneously (B(i)). Transfer impedances were recorded with injection of current through different electrode pairs for 30 seizures; locations of and the distance between EIT electrodes were varied to ensure adequate sampling of cortical tissue (B(ii)). A 7-min rest period between stimulation series prevented neocortical kindling effects during imaging protocols.

Seizures were induced on demand by electrical stimulation of the sensorimotor cortex through two electrodes on the 57-electrode array, with a centre-to-centre distance of 2.4 mm, using a Keithley 6221 current source (Keithley Instruments Ltd., Bracknell, UK) (Supplementary Information 1.4). 5-s trains of biphasic, charge-balanced square-wave pulses with a 1 ms pulse width were delivered at 100 Hz (Fig. 1A). Current of 2.0 ± 0.8 mA (*n* = 168 seizures, *N* = 5 rats) was variably applied to produce a consistent ECoG pattern of rhythmic SWDs. An inter-stimulus interval of 7 min prevented kindling of neural circuits over time to ensure that seizure patterns remained consistent during experiments (Nelson et al., 2010). If motor manifestations of seizures were severe enough to produce artefacts in ECoG recordings, rats were paralysed prior to commencing EIT measurements with administered pancuronium bromide (1 mg/kg i.v.). All procedures were performed on a vibration isolated table (Thorlabs Inc., Newton, NJ, USA).

### 2.3 EIT hardware, data acquisition and protocols

The ScouseTom EIT system, comprising the BrainVision actiCHamp 128-channel EEG amplifier (Brain Vision LLC, Cary, NC) and Keithley 6221 current source (Keithley Instruments Ltd., Bracknell, UK), was used to obtain ECoG and tissue impedance recordings simultaneously (Avery et al., 2017). Recordings were made from 64 channels and digitised at a sampling frequency of 50 kHz. For all EIT recordings, a constant sinusoidal current was injected through a single pair of electrodes on the 57-electrode array at a carrier frequency of 1.725 kHz and an amplitude of 50 μA; independent voltage measurements for the resulting current pattern were obtained from the remaining electrodes on the array. Each impedance recording contained the seizure, in addition to baseline periods of ≥10 s prior to cortical stimulation and ≥30 s after the last ictal discharge.

A full EIT protocol for imaging cortical epileptiform activity comprised up to 30 independent impedance measurements and a different electrode pair for current injection was used for each of 30 seizures (Fig. 1B). The current-injecting electrodes in the EIT protocol were separated by 2.4–18.6 mm. Since the tissue penetrating depth of the injected current is dependent on the inter-electrode distance, the electrode-addressing sequence was determined using mathematical modelling to generate the smallest set of orthogonal current patterns needed to provide uniform sensitivity to impedance changes throughout the underlying cortical tissue in the hemisphere of interest. This provided a depth sensitivity of 2 mm from the cortical surface.

### 2.4 Experimental controls

Control recordings were made to validate that the cause of the observed impedance changes was in fact the induced epileptiform activity. First, the 10-s baseline impedance recording prior to electrically stimulating the sensorimotor cortex confirmed that the measured impedance change was not due to resting cortical activity. This was further verified by obtaining at least three impedance recordings at the beginning of each EIT protocol in which cortical tissue remote from the sensorimotor region, so as not to induce seizures, was stimulated with the same electrical parameters as those corresponding to the seizure threshold. The latter control also demonstrated that these changes were not due to the immediate, acute effect of current-induced excitation of local groups of cortical pyramidal neurons before they attain the level of hypersynchronisation which underlies epileptiform activity. Lastly, to eliminate the possibility of the impedance changes being due to an artefact caused by the cortical stimulation procedure itself, a full EIT protocol was performed in two dead rats. Furthermore, ECoG traces of induced seizures were visually assessed in the absence of EIT recordings to confirm that continuous EIT current injection did not alter the electrographic pattern of ictal discharges.

### 2.5 Analysis of ECoG and impedance data

The recorded raw voltage measurements contained both the ECoG and impedance signals; different filter settings were utilised to extract each of these in turn. The ECoG signal was obtained by application of Butterworth filters at 1 kHz (low-pass, fifth order) and 1 Hz (high-pass, first order), in addition to a 50 Hz IIR notch filter. To extract the impedance signal, the recording was filtered and demodulated with a bandwidth of ±500 Hz around the carrier frequency of 1.725 kHz (fifth-order Butterworth), giving a temporal resolution of 2 ms.

The channel displaying the highest-amplitude ictal discharges in the filtered ECoG traces, which remained consistent across all seizures and animals, was selected as the trigger signal for subsequent spike detection and sorting to enable averaging of SWD-related impedance changes within seizures. This relies on the assumption that SWDs are sufficiently reproducible, which was validated using an automated neuronal spike classification algorithm (Quiroga et al., 2004; Supplementary Information 1.5). The SWD, the only pattern of epileptiform activity that was seen consistently across all seizures in all rats, was defined as the combination of a single sharp spike, <70 ms in duration and with a peak-peak amplitude of ±2 mV, followed by a slow wave component lasting around 100 ms. Trigger markers were set at the peak of the spike component of all detected SWDs and verified by manual inspection (Fig. 1A). Only individual SWDs that lay within ±3 standard deviations (SDs) of the mean trace for all detected SWDs in a given seizure were used for averaging.

The demodulated impedance signals from all channels for each seizure were aligned with respect to the trigger markers. For each electrode channel, the SWD-related impedance change (dZ) was averaged within recordings, resulting in a mean dZ trace per seizure. A 150-ms temporal window around the trigger marker which encompassed the entire SWD was used for averaging. The baseline dZ for each channel was defined as the mean amplitude during the first 10 ms and time points with significant activity were identified across all channels by comparing the dZ value with the baseline dZ using a paired *t*-test, at a significance level of α = 0.01. Current-injecting and excessively noisy channels were rejected from further analysis; the latter were defined as channels with boundary voltages <100 μV. Channels were also rejected by thresholding according to the baseline SD: within the time period of significant activity, significant channels were defined as those with dZ greater than three times the baseline SD and were preserved. dZ measurements from remaining channels, typically 90% of the total, were collated across seizures to produce an image of an individual SWD. All data are presented as mean ± SD.

### 2.6 Topographic reconstruction of cortical impedance changes

EIT images of the SWD were produced using ≤1650 processed voltages, which comprised ≤55 voltage measurements (57 electrode channels minus current-injecting and excessively noisy channels) for each of 30 independent current injections. The forward solution involves prediction of boundary voltages resulting from a defined current injection protocol. This was calculated for a given EIT protocol on an anatomically realistic finite element method (FEM) mesh of the rat brain, generated from MRI images obtained in 21 adult Sprague-Dawley rats (Nie et al., 2013) and comprising 2.9 million tetrahedral elements, using the PEITS forward solver with the complete electrode model (Boverman et al., 2007; Jehl et al., 2015a). Measurements were obtained with respect to a reference electrode, 9 mm in diameter, placed over the dorsal surface of the cerebellum. Tissue conductivity was assumed to be isotropic throughout the cerebral grey matter (0.3 Sm^−1^) and white matter (0.15 Sm^−1^), and the conductivity of cerebrospinal fluid was set at 1.79 Sm^−1^ (Ranck, 1963; Baumann et al., 1997; Latikka et al., 2001) (compiled from literature by Horesh (2006)); summarised by Jehl et al. (2015b))). An inversion of the resulting Jacobian matrix was used to solve the inverse problem. To prevent the inverse crime and reduce computational time (Lionheart, 2004), reconstructions were performed on a coarse hexahedral mesh containing approximately 80,000 elements, 300 μm in length. Zeroth-order Tikhonov regularisation with noise-based correction was applied after selection of the regularisation parameter through generalized cross-validation (Tikhonov et al., 1995; Aristovich et al., 2014). Images were reconstructed at 1 ms time-steps for the entire temporal window of averaged SWD-related impedance changes.

The reconstructed conductivity changes in each hexahedral voxel were corrected using a previously described noise-based correction approach and expressed as t-score (δσ) for visualization (Aristovich et al., 2014). This involves dividing the reconstructed conductivity values by the computed SD of the estimated conductivity change, due to random Gaussian noise in the voltage measurements, for each hexahedron (Aristovich et al., 2014).

### 2.7 Analysis of reconstructed images

The reproducibility of images was assessed quantitively with population statistics across all animals using a binomial mask. Active voxels were defined as those with those with δσ ≥ 3 (*p* < 0.01) and the volume of active voxels with *p* < 0.03125 (0.5^5^) across all five rats was visualised. Images were also qualitatively evaluated with respect to a detailed anatomical atlas of the rat brain (Paxinos and Watson, 2013) to determine brain regions implicated in the reconstructed SWD-related activity.

To assess the possibility of determining the propagation of spike-wave activity through cortical layers, centre-of-mass analysis was conducted after reconstructing images with a finer hexahedral mesh in which elements were 150 μm in length. The centre-of-mass of activity was calculated by determining the largest connected cluster of active voxels in 4D at a threshold of 50% of the maximal δσ (full-width at half maximum [FWHM]). Significant time points were defined as those with active voxels ≥20% of the maximal δσ. The resulting values were superimposed on the mesh for qualitative visual inspection. Centre-of-mass analysis could only be performed in 4 rats as one EIT image contained a large artefact reconstructed around the ground electrode which would have considerably distorted the trajectory of the centres of activated clusters.

## 3 Results

### 3.1 Electrographic features of seizures induced by the cortical stimulation model of epilepsy

**Fig. 2:**
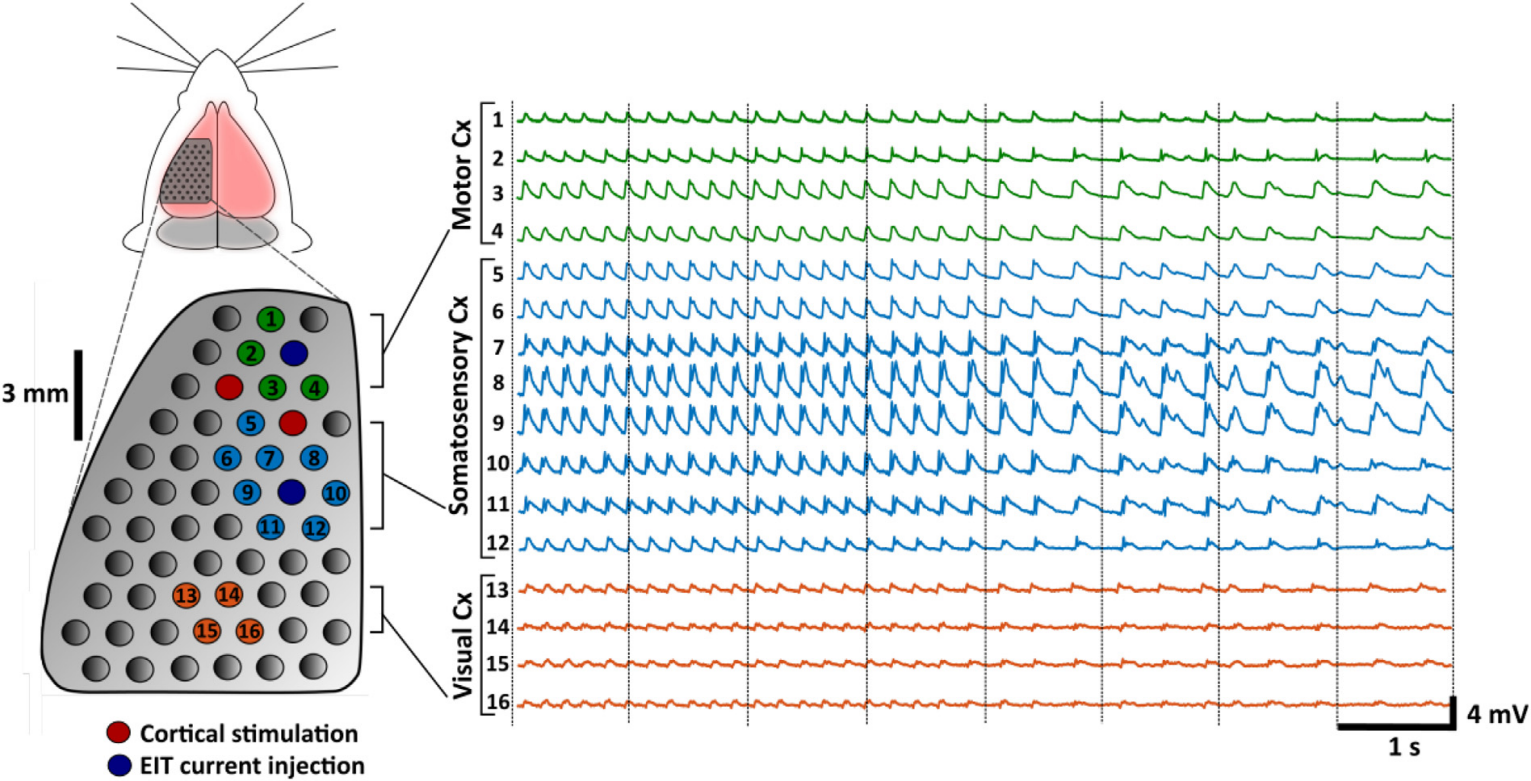
ECoG recording from a typical seizure induced by the cortical stimulation model of focal spike-wave activity. An 8-s epoch of a representative seizure is displayed beginning at 2 s after the end of cortical stimulation. For the purpose of clarity, ECoG traces are presented only from a selection of electrodes on the 57-electrode epicortical array; these covered three distinct regions of the cortex: motor (top), somatosensory (middle) and visual (bottom). Electrode pairs for stimulating the sensorimotor cortex (red) and EIT current injection (blue) are indicated. The epoch begins with 5 Hz SWDs with subsequent slowing to 2–3 Hz as the seizure progresses; SWDs in the first 4 s were classified into a single group by the automated spike classification algorithm used and so were deemed to be repeatable enough to average together. The focus of this spike-and-wave activity is in the primary somatosensory cortex, approximately 3 mm posterior to the site of stimulation, and the amplitude of ictal discharges decreases as the spiking activity emanates from this location.

Seizures comprised a characteristic pattern of focal, rhythmic SWDs at 2–5 Hz and minimum peak-to-peak amplitude of 2 mV immediately after stimulation (Fig. 2), occasionally followed by other more variable epileptic discharges including sharp waves and polyspike complexes. Mean seizure duration and number of SWDs detected and used for averaging were 16.5 ± 5.3 s and 46 ± 19, respectively (*n* = 168 seizures, *N* = 5 rats). Tonic posturing occurred during cortical stimulation and seizures were accompanied by facial and contralateral forelimb clonus in unparalysed rats.

### 3.2 Characterising the impedance response to ictal SWDs

**Fig. 3:**
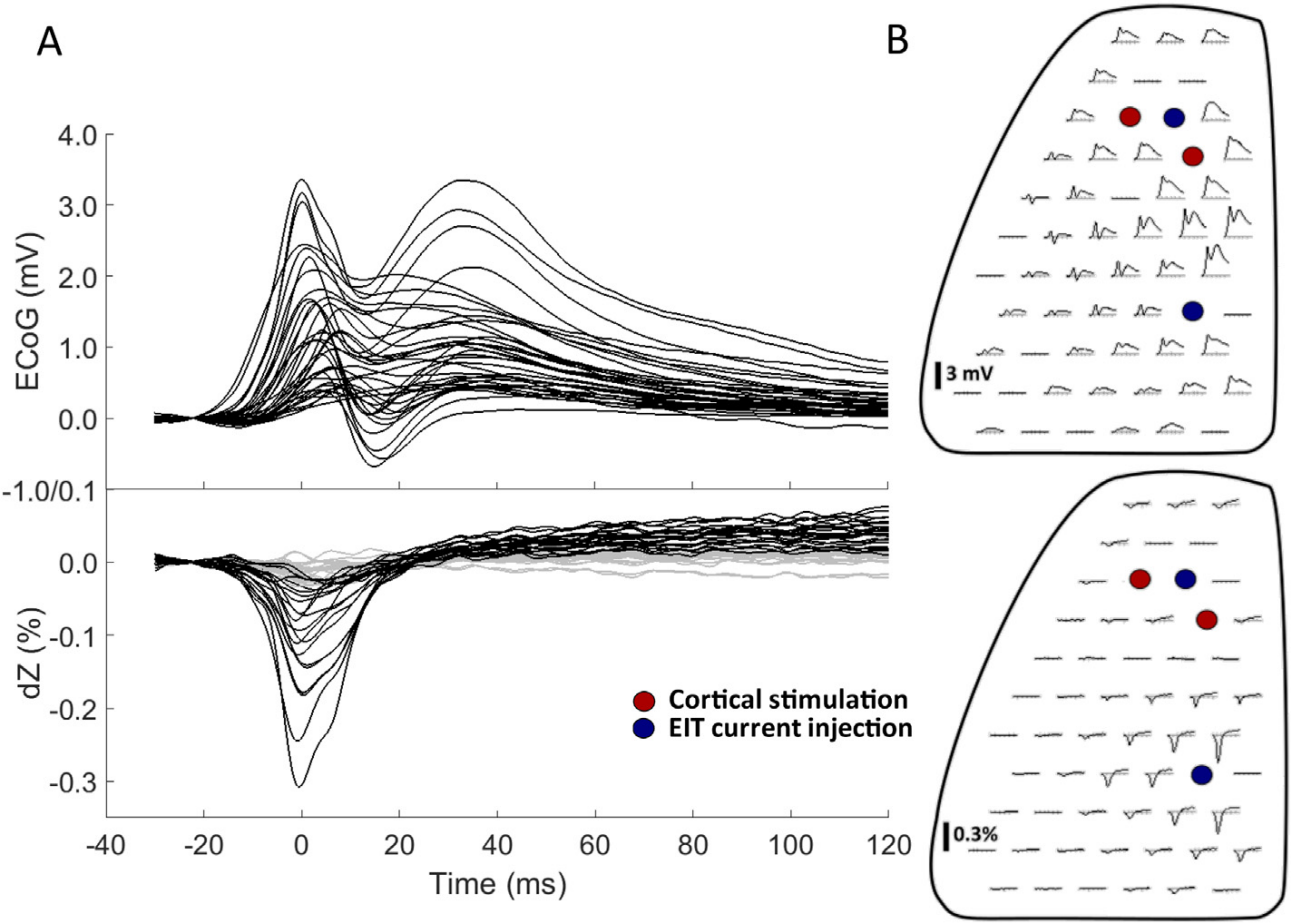
Representative example of the spatial and temporal features of the impedance (dZ) response to SWDs. A, ECoG (top) and impedance (bottom) recordings of an ictal SWD from the 57-electrode epicortical array, averaged across a single seizure. Non-significant impedance traces, defined as those with a maximum amplitude <3 SD from the baseline mean are plotted in grey. B, Spatial arrangement of ECoG (top) and impedance (bottom) traces across the 57-electrode epicortical array. Channels with stimulation artefacts or excessive noise have been removed and are shown in the array as flat traces. Electrode pairs for stimulating the sensorimotor cortex (red) and EIT current injection (blue) are also indicated. The largest impedance traces were recorded from electrodes around one of the current-injecting points as this is where current density, and thus sensitivity to impedance changes, was at its highest.

Following spike detection and classification, the ECoG and impedance data for individual SWDs within each seizure were averaged together. Averaged dZ responses to ictal SWDs were characterised by a consistent impedance decrease of −0.31 ± 0.06%, lasting ~20 ms, in phase with the spike component in the ECoG and then a more variable increase of 0.16 ± 0.19% associated with the wave component, lasting >100 ms (*n* = 168 seizures, 5 rats, Fig. 3). The location of the maximum dZ for each EIT recording depended on the site of current-injecting electrodes used to obtain it, which determined the current density and sensitivity through the underlying cortical tissue (Supplementary Fig. S2).

### 3.3 EIT images of ictal SWDs and statistical analysis

**Fig. 4:**
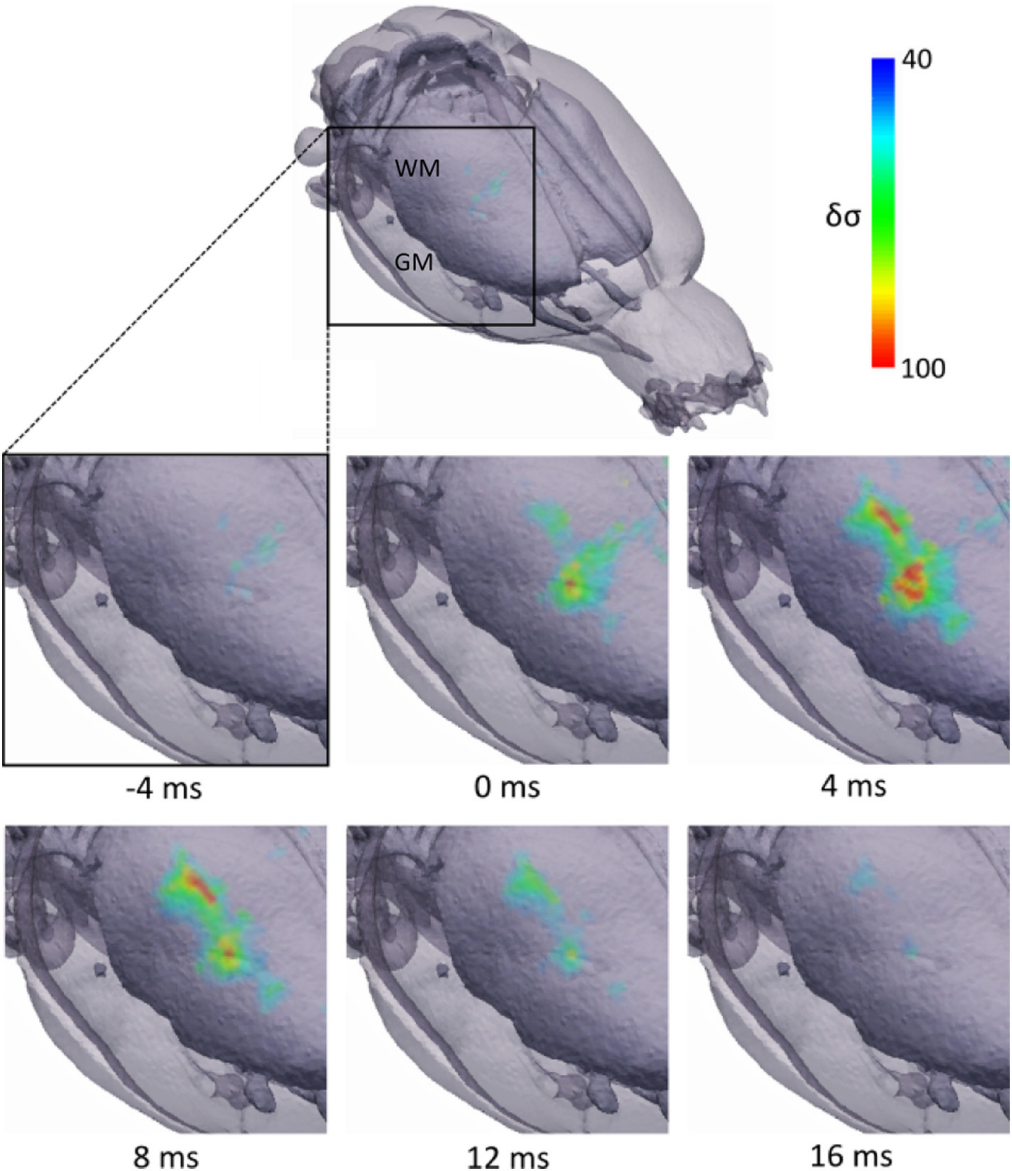
EIT image showing fast electrical impedance changes during the spike component of an averaged ictal SWD. The sequence of images, every 4 ms over a total time period of 21 ms, reveal an early focus of activity confined to the whisker barrel area in the primary somatosensory cortex, slightly posterior to the sensorimotor region which was electrically stimulated to induce seizures. A subsequent posterior and lateral spread of activity is observed over the following period while the activated volume reaches a maximum around 4 ms, before reducing in size. Time is given with respect to the peak EEG amplitude of the ictal SWD and δσ represents t-score of conductivity changes. GM, grey matter; WM, white matter.

**Fig. 5:**
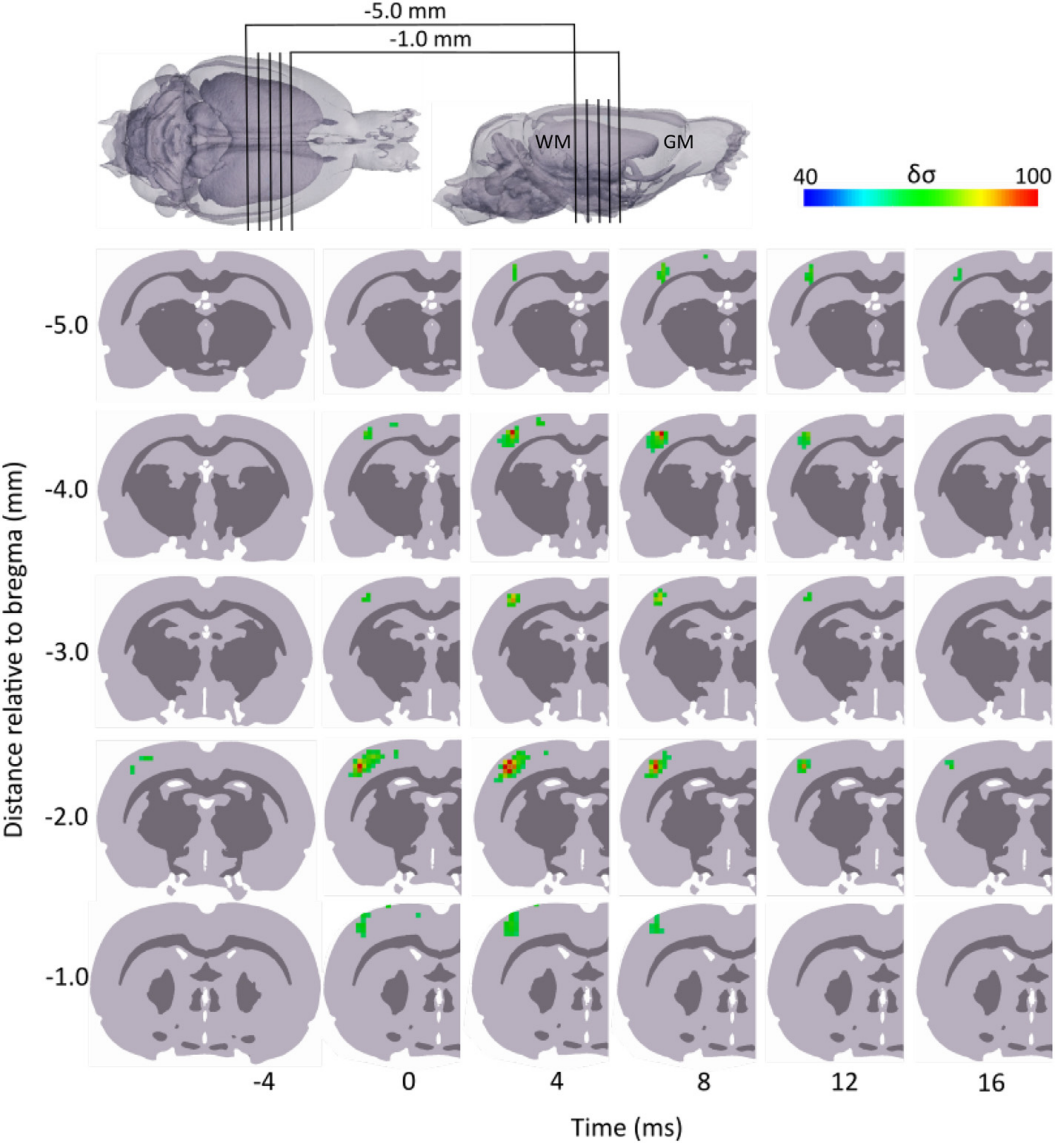
Anteroposterior and dorsoventral propagation of reconstructed SWD-related impedance changes. Coronal slices were obtained for six time points from −4 to 16 ms, with respect to the peak EEG amplitude of the ictal SWD, at five distances in the anterioposterior direction relative to bregma: −1.0, −2.0, −3.0, −4.0 and − 5.0 mm. Slices obtained at −2.0 and − 4.0 mm transect the central portion of two distinct regions of maximal δσ. Movement of fast electrical activity in the posterior direction can be observed as the maximal δσ in anterior slices (−1.0 and − 2.0 mm) is achieved at an earlier time point (4 ms) compared to the three more posterior slices (−3.0, −4.0 and − 5.0 mm), in which the largest volume of cortical tissue is activated at 8 ms. Simultaneously, a general shift of δσ in the dorsovental direction can be observed over time: whereas early activity is concentrated in the superficial half of the cortex, the ensuing time period sees its gradual propagation to deeper cortical tissue. δσ represents t-score of conductivity changes. GM, grey matter; WM, white matter.

Each processed dataset was reconstructed into an EIT image of a single averaged ictal SWD. EIT images were reconstructed every 1 ms and spanned −20 to 20 ms, where 0 ms corresponds to the peak amplitude of the spike component in the ECoG trace; this time frame encompassed the entire spike phase, in which significant δσ were observed. Significant dZ during the spike phase arose in the whisker barrel representation in the primary somatosensory cortex, before encompassing a larger volume as the maximum ECoG and dZ values were reached (Fig. 4, *n* = 168 seizures, *N* = 5 rats). The volume of active voxels then gradually decreased until the end of the spike. Visual inspection indicated that the activity propagated in the lateral and posterior direction from its initial focus in the barrel cortex, and exhibited a bimodal spatial profile of activation after 0 ms, with the centres of the two maximally activated regions localised to 2.0 and 4.0 mm posterior to bregma (Fig. 4, Fig. 5). Activity also appeared to propagate ventrally during its movement in the posterior direction (Fig. 4).

**Fig. 6:**
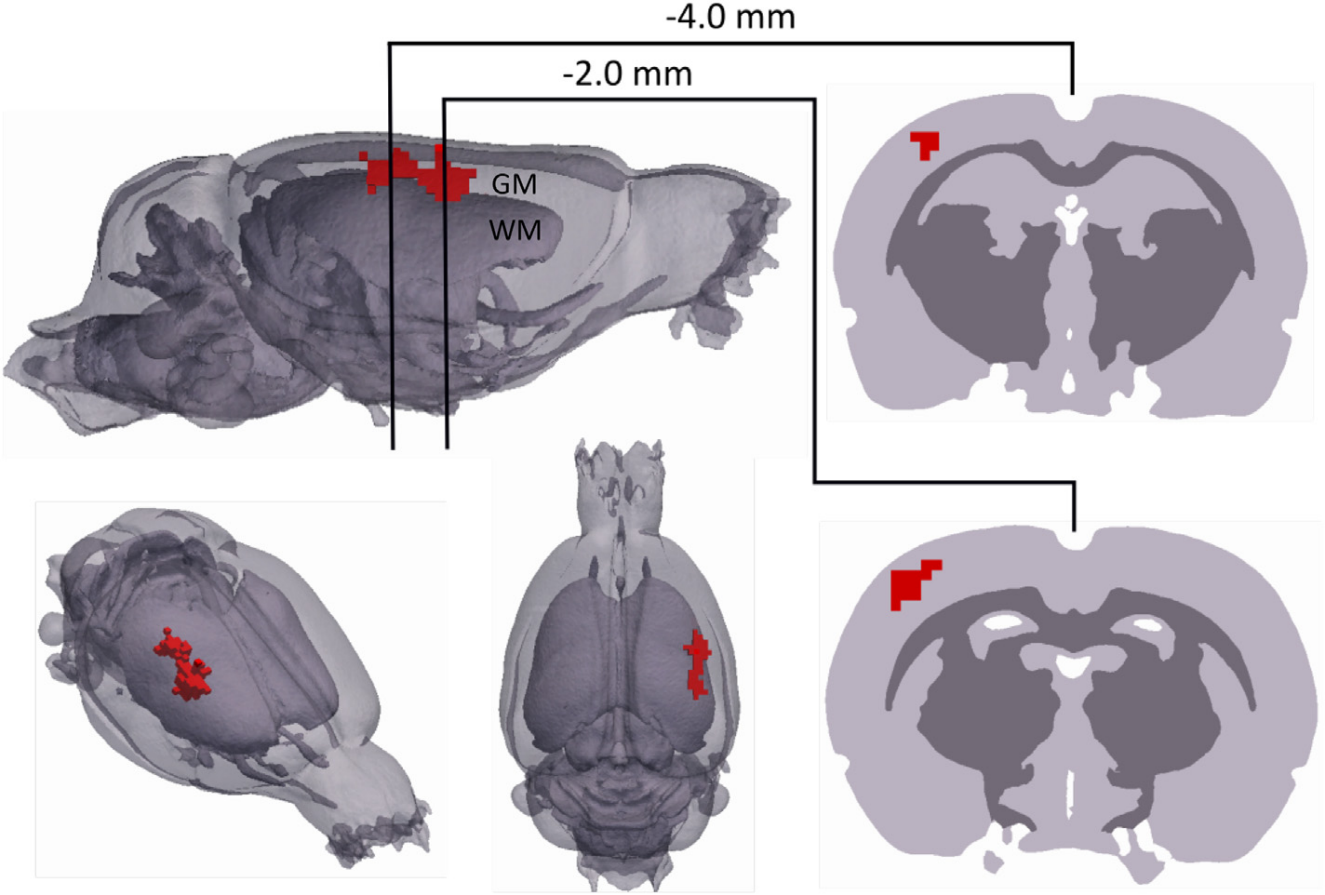
Population statistics of EIT images. Sagittal, anterolateral and dorsal views (left) of significant active voxels (red) in the reconstructed δσ across rats at 4 ms relative to the peak EEG amplitude of the ictal SWD (p < 0.03125, N = 5). Two coronal slices which transect the maximally activated areas of cortical tissue, obtained at 2.0 and 4.0 mm posterior to bregma, are also provided (right). GM, grey matter; WM, white matter.

Application of a binomial mask showed a spatially distinct region of common active voxels across rats during the spike phase which matched the location of reconstructed δσ for individual rats (Fig. 6, *p* < 0.03125, *n* = 168 seizures, *N* = 5 rats). Control recordings further verified that the observed activity was not artefactual; baseline (N = 5 rats) and dead controls (*N* = 2 rats) showed no significant focal or diffuse activity when processed and reconstructed with the same methods as those for evoked seizures.

### 3.4 Translaminar features of SWD-related impedance changes

**Fig. 7:**
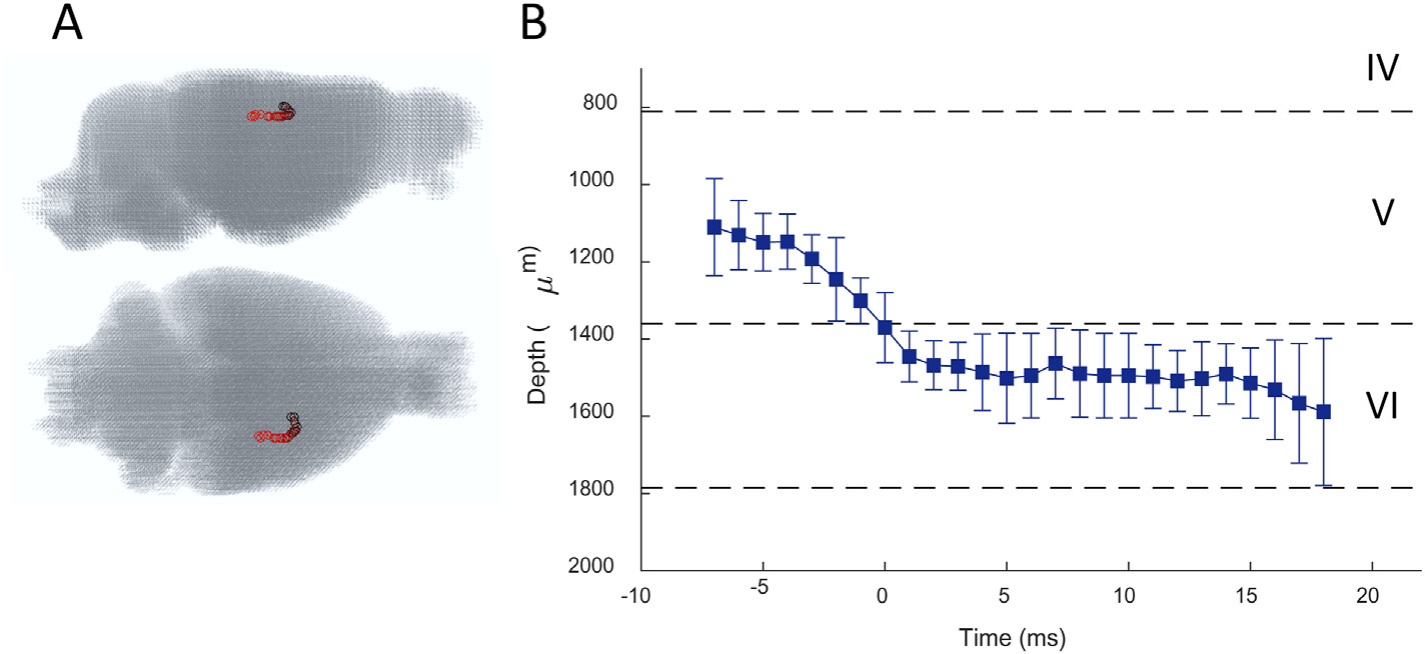
Centre-of-mass analysis of reconstructed fast neural δσ during spike phase of SWD. A, Representative example of lateral (top) and dorsal (bottom) views of the centre-of-mass of reconstructed δσ at each significant time frame superimposed on the mesh, demonstrating that the activity follows a distinct trajectory. B, Translaminar depth of the centre-of-mass of activity is plotted against time, with respect to cortical layers IV-V in the facial somatosensory cortex. Boundaries of cortical layers were calculated from average laminar thicknesses in the rat barrel cortex, as described previously (Di et al., 1990; Paxinos and Watson, 2013). Values of centre-of-mass depth are means, with error bars representing 99% confidence intervals (*n* = 135 seizures, *N* = 4 rats). Time is given with respect to the peak EEG amplitude of the ictal SWD.

Centre-of-mass analysis demonstrated that onset of activity occurred at −7 ms, with respect to the peak EEG amplitude of the SWD, at a depth of 1110 ± 84 μm beneath the pial surface, corresponding to Layer V of the whisker barrel cortex (Fig. 7, *n* = 135 seizures, *N* = 4 rats). The centre of mass then propagated ventrally with time, entering Layer VI between −1 and 1 ms, simultaneously to its spread in the posterior direction. It remained in Layer VI until the last significant time point, at which point δσ was localised to a laminar depth of 1590 ± 127 μm. These findings demonstrate that the foci of SWD-related fast neural impedance changes are spatially confined to deep layers of the primary somatosensory cortex.

## 4 Discussion

Here, we have used EIT to characterise the propagation pattern of the impedance response to focal ictal spike-and-wave activity, induced by the cortical stimulation model of epilepsy in the anaesthetised rat, using non-penetrating epicortical electrodes. Averaged SWDs consistently displayed a sharp impedance decrease, which can be attributed to hypersynchronous depolarisation of local populations of cortical pyramidal neurons, due to alterations in sodium and calcium conductance (Blumenfeld, 2005). Reproducible tomographic images of fast electrical activity during the spike phase of ictal SWDs are presented for the first time; these were generated from dZ recordings with a spatial and temporal resolution of 300 μm and ≤ 2 ms, respectively. Images reveal an early focus of neural activity to be spatially confined to the whisker barrel cortex. It then increased in size in phase with the EEG amplitude of the spike, and propagated posterolaterally and ventrally within the facial somatosensory cortex. The site of maximal reconstructed impedance changes typically matched the ictal focus, as determined by ECoG recordings. Fast neural EIT has therefore been shown to deliver a reliable method capable of imaging impedance changes associated with neuronal depolarisation in ictal events to improve understanding of their pathophysiology, which may ultimately aid the development of targeted therapies for preventing or treating neocortical epilepsies.

### 4.1 Technical considerations

The ictal SWDs imaged in this work were induced by the cortical stimulation model of epilepsy, in which electrical stimulation of the sensorimotor cortex results in an immediate run of epileptiform discharges. As such, the timing of ictal events could be tightly regulated, enabling total control of the protocol used to obtain impedance measurements for imaging. The optimised stimulus parameters successfully generated seizures with stereotypical electrographic features in all rats, and the repetitive nature of individual SWDs within and across seizures enabled averaging of the associated dZ to increase SNR for imaging. An initial concern was that variance in SWDs over the course of the seizure would have distorted the averaged signal used for reconstruction. However, strict criteria were used to identify SWDs that were similar based on waveform, amplitude, duration and interspike interval using automated wavelet transforms and paramagnetic clustering (Quiroga et al., 2004). In addition, detected SWDs were further evaluated to preserve only those that exhibited a high degree of electrographic repeatability, and so it could be assumed that they were induced by activation of the same neural circuitry, allowing for their averaging. The current work has thus validated the cortical stimulation model of epilepsy as a reliable means for inducing focal ictal SWDs in an acute setting to evaluate the feasibility of using EIT to image this activity.

Visual assessment of reconstructed images revealed a subtle movement of activity in the ventral direction over time; this was particularly evident at the two distinct regions of maximal activity (−2.0 and −4.0 mm from bregma). Centre-of-mass analysis on a finer hexahedral mesh (150-μm elements) was conducted to quantitatively assess the propagation pattern of this activity along the dorsoventral axis, although this approach was limited in several respects. First, the centre-of-mass was calculated in 4D to ensure that the propagation volumes of both of the two distinct maximally activated clusters of activity were taken into consideration to arrive at a weighted centre-of-mass position. However, because the imaged activity was bimodal in nature, the computed centre-of-mass at any time point did not indicate the absolute location of either of the sites of maximal δσ. Rather, it's purpose was to serve as a succinct visual summary of the images displaying the overall trend in depth of activity when considering all active voxels at a given time point during the ictal spike. Secondly, although the centre-of-mass of activity was spatially confined to the infragranular layers (V and VI) of the somatosensory cortex, the actual activated mass volume may still transcend the more superficial cortical layers. Further, the mapping of activity foci to individual cortical layers was not validated with local field potential (LFP) recordings using an intracortical microelectrode array, as this may have corrupted seizure generation, and so cannot be definitively concluded.

In the EIT images obtained, the propagation of SWD-related fast electrical activity was restricted to the cerebral cortex. Although many studies in rat models of absence epilepsy have shown that a cortical focus is dominant in initiating the hypersynchronous neuronal firing which underlies cortical spike-and-wave activity (Meeren et al., 2002; Meeren et al., 2005; Polack et al., 2007; Polack et al., 2009), the ventrobasal nuclei of the thalamus are also known to be implicated to some extent (Polack et al., 2007). Thus, it was desirable to determine the involvement of the thalamus in the expression of these paroxysmal discharges for definitive elucidation of their spatiotemporal dynamics. However, the SNR achieved by fast neural EIT using epicortical arrays with the described electrode geometry and EIT protocol yielded sufficient resolution to a penetration depth of ~2 mm. As such, this study was restricted to imaging conductivity changes in the cerebral cortex, although it still provides novel insights into the trajectory of SWDs over large volumes of the cortex not previously achieved with other techniques. Modelling studies have indicated that a spatial resolution of 200 μm can be obtained if electrodes, spaced ≤2 mm apart, are present in cerebral tissue enclosing the subcortical region of interest, namely, the ventrobasal thalamic nuclei. We are currently developing methods for imaging thalamic activity contemporaneously with the cortex using LFP depth electrodes placed around the thalamus.

### 4.2 Comparison of findings to literature and implications for understanding mechanisms of ictal SWDs

This fast, transient decrease in cortical tissue impedance observed during the spike phase of ictal SWDs is comparable to that which has been previously described during interictal spikes induced by chemical models of epilepsy (Vongerichten et al., 2016). Whereas Vongerichten et al. also reported a subsequent larger dZ increase during interictal spikes, no such significant activity could be reconstructed during the wave component of SWDs in the current study. This may be explained by the fact that interictal discharges, despite being widely accepted as a diagnostic indicator of epilepsy, are thought to be mediated by distinct pathophysiological mechanisms to ictal discharges and may not be indicative of the seizure onset zone itself (McBride et al., 1991; Stafstrom, 2007; de Curtis et al., 2012).

The localisation of reconstructed fast neural impedance changes to the facial somatosensory cortex during ictal discharges is in agreement with previous studies in WAG/Rij and GAERS rats, genetic models of absence epilepsy, which have demonstrated the importance of facial projections in the somatosensory cortex for driving the generation of SWDs (Meeren et al., 2002; Sitnikova and van Luijetelaar, 2004; Meeren et al., 2005; Polack et al., 2007; Polack et al., 2009). Since it was the sensorimotor cortex that was electrically stimulated to induce seizures, location of the initial ictal focus in the whisker barrel subfield also suggests that functional connectivity between these two cortical regions may be involved in SWD generation. Centre-of-mass analysis of reconstructed images indicated a ventral propagation of activity foci over time and suggested that these foci are located in the deep facial somatosensory cortex. This is consistent with previously published findings which, using *in vivo* intracellular recordings, have shown an essential role for deep-layer cortical neurons in initiation of these paroxysmal discharges (Polack et al., 2007; Polack et al., 2009).

### 4.3 Potential clinical applications for fast neural EIT

The proposed method may be used in patients with intractable focal epilepsies undergoing presurgical evaluation with subdural grid electrodes, in conjunction with source localisation based on ECoG recordings (Dümpelmann et al., 2009; Dümpelmann et al., 2012). The critical limitation of EIT relates to the need for averaging due to the low magnitude of the impedance signal measured from the cortical surface. The SNR of ECoG recordings, on the other hand, is considerably higher and thus permits real-time signal classification. However, more independent measurements can be obtained with EIT than ECoG using the same number of electrodes since transfer impedances are recorded for every independent current injecting electrode pair in the EIT protocol, which increases the spatiotemporal information utilised for image reconstruction. Additionally, EIT has a unique solution to the inverse problem (Somersalo et al., 1992), unlike source reconstruction methods, in which multiple current models may fit the recorded data. Therefore, it would be advantageous to utilise both methods simultaneously in these clinical cases to aid localisation of the ictal onset zone with improved spatial accuracy (Witkowska-Wrobel et al., 2018).

The presented approach holds potential to image a variety of electroencephalographic seizure patterns, provided that the electrode arrangement for EIT current injection offers an adequate sensitivity for imaging in the region of interest. At present, the depth penetration of EIT with epicortical electrode arrays extends to the dorsal hippocampus (Faulkner et al., 2018); this setup can therefore be used to image seizure activity throughout the cortex and in superficial hippocampal regions. The initial discharges within the seizure are of particular importance for localising the ictal onset zone; it would thus be desirable to specifically image the seizure onset pattern. However, our methods required averaging of ≥30 repeatable ictal discharges due to the low SNR of the fast neural impedance changes associated with individual SWDs, and thus relied on a relatively stable state of electrographic activity during the seizure. Therefore, this approach as it stands cannot be used to image only the initial discharges within seizures which constitute the seizure onset pattern, particularly if they are of low amplitude and have a high degree of variance. Future developments in hardware and signal processing that improve SNR may extend the capabilities of EIT to image different seizure onset patterns, which would vastly improve its clinical potential.

### 4.4 Conclusion

The current work has shown that EIT can be employed for imaging fast electrical activity associated with epileptic discharges at a resolution of 300 μm and ≤2 ms in the rat cerebral cortex using non-penetrating surface electrodes. In addition to supporting the cortical focus theory of ictal spike-and-wave activity, these results have demonstrated the potential of EIT for providing novel insights into the spatiotemporal dynamics of aberrant neuronal firing during epilepsy. In the future, it will be of interest to investigate the temporal characteristics of thalamic activation with respect to the imaged propagation pattern of ictal SWDs using EIT with epicortical and depth electrodes. Additionally, since the cortical stimulation model used here lacks neuronal cell-type specificity, the direct clinical usefulness of this methodology for aiding localisation of ictal foci can be assessed by utilising an epilepsy model which provides a more accurate representation of the pathophysiology of human epilepsies. An example of such a model includes intrahippocampal administration of kainic acid in rats, which generates well-documented, neuropathological correlates of clinical temporal lobe epilepsy such as neuronal loss and mossy fiber sprouting (Ben-Ari, 1985; Tauc and JV, 1985; Okazaki et al., 1995). If fast neural impedance changes associated with more electrographically variable, spontaneous ictal events with different onset patterns induced by such experimental models can be successfully imaged, this will establish EIT as a valid future neuroimaging tool for the clinical detection and localisation of ictal events.

## Appendix A Supplementary data

Supplementary material Image 1
