# Supplementary material for: Imaging fast electrical activity in the brain during ictal epileptiform discharges with electrical impedance tomography
Source: Neuroimage Clin. 2018 Sep 5;20:674–84. doi: 10.1016/j.nicl.2018.09.004 (PMC6140294; doi:10.1016/j.nicl.2018.09.004)
Supplement: Supplementary file 1 — Supplementary material [file mmc1.docx]

Imaging fast electrical activity in the brain during ictal epileptiform discharges with electrical impedance tomography

Sana Hannan ^a*^, Mayo Faulkner ^a^, Kirill Aristovich ^a^, James Avery ^a^, Matthew Walker ^b^, David Holder ^a^
^a^ Department of Medical Physics and Biomedical Engineering, University College London, UK
^b^ Institute of Neurology, University College London, UK
*Email:* sana.hannan.14@ucl.ac.uk*

**SUPPLEMENTARY INFORMATION**

1. *Supplementary Methods*
   1. **57-electrode planar epicortical array**

Epicortical electrode arrays used for ECoG and impedance measurements were custom-designed and fabricated in-house using laser-cut platinised stainless-steel foil (12.5 µm thick) sandwiched between two layers of medical grade silicone rubber as a base for insulation (Koronfel, et al., 2014). 57 electrodes within the array, each 0.6 mm in diameter, were arranged hexagonally with a centre-to-centre distance of 1.2 mm between adjacent electrodes (**Supplementary Fig. 1**; Koronfel, et al., 2014).


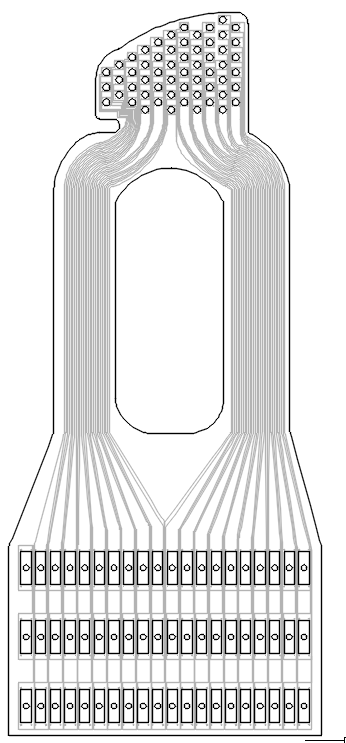


*

A


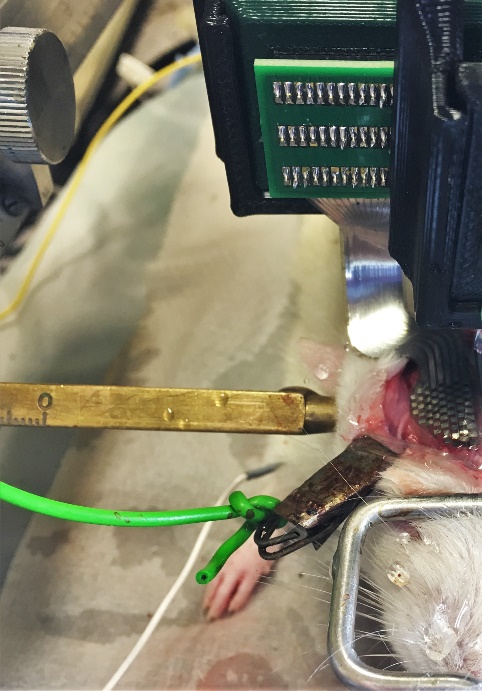


**

C


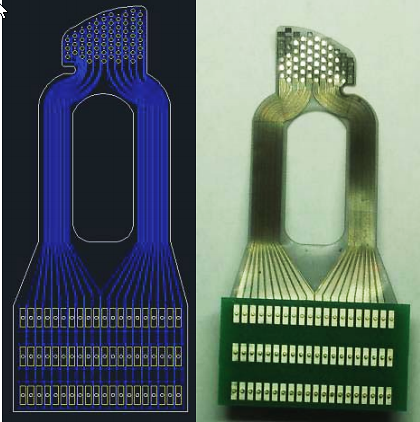


9 mm

B

Electrode tracks

Connector


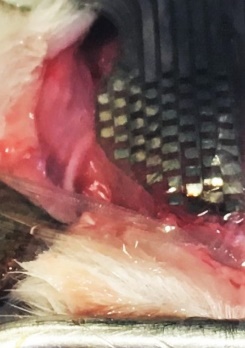


**


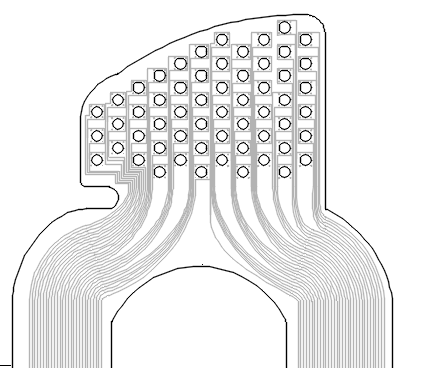


*

600-µm platinised stainless steel electrode contact

**Supplementary Figure S1. 57-electrode planar epicortical array.** A CAD drawing (A) and photograph of the fabricated array (B); each electrode contact was 600 µm in diameter. (C) A photograph of the electrode array placed on the right hemisphere of the brain of a Sprague-Dawley rat following craniotomy. Magnifications showing details of the hexagonal electrode arrangement in A (*) and C (**) are provided.

The thickness of the saline layer at the cortical tissue-electrode interface was controlled by the nature of the conformal planar electrode array and was taken into consideration during its design. Due to the method of fabrication of the array, the hydrophilic insulating silicone rubber was in direct contact with the cortex while the electrode contacts were slightly recessed into the rubber. As such, the saline layer on the interface matched the electrode geometry and was consistent across experiments.

- 1. **Coregistration of electrodes between experiment and model**

The planar epicortical electrode array, which provided extensive coverage of the neocortical surface of one hemisphere, was designed such that the deformed flexible silicone rubber was conformal to the curved brain. The trapezoidal perimeter of the craniotomy, which followed the shape of the array, was marked out from bregma, an anatomical reference point on the skull surface, in the same position for all rats using high-precision stereotaxic micromanipulators (SM-15; Narishige International Ltd, London, UK). Thus, because the craniotomy was large enough to only fit the electrode array, the electrodes were expected to automatically align on implantation to match their positions in the model. To confirm this, the array was visually inspected under light microscopy to ensure that the centres of all electrode contacts on the fourth most anterior electrode row were in alignment with the bregma point. Therefore, because we specifically aimed to align the centres of these electrodes with bregma and did not proceed with the experiment until this was ensured, the experiment-to-model coregistration error for an individual animal was approximated to be ≤0.3 mm, the radius of a single electrode contact.

- 1. **Choice of epilepsy model: the cortical electrical stimulation model**

*In vivo* experimental models of chronic epilepsy or acute seizures may be induced by chemoconvulsants, electrical stimuli or traumatic brain injury (Fisher, 1989). For this work, we chose a model in which seizures could be reproducibly elicited using electrical stimulation of the rat cerebral cortex. This is usually performed by electrical stimulation of the sensorimotor area with a train of square-wave pulses lasting up to 15 s (Kubová & Mares, 1995; Kubová, et al., 1996; Haugvicová, et al., 2002; Nelson, et al., 2010). Advantages of this model include the following.

1. It enables induction of epileptiform events on demand, typically immediately after the end of the stimulation period. Whereas obtaining impedance measurements of spontaneous ictal events is typically challenging due to their unpredictable nature, this model would enable complete control of the EIT current injection protocol for cortical imaging. Its ability to induce frequent recurrent ictal events also maximises the amount of impedance data obtained from a single animal for more accurate image reconstruction.
2. Contrary to the effects of injecting a chemoconvulsant into the cortex, continuous or intermittent electrical stimulation of cortical tissue does not induce irreversible plastic changes to local neural circuits at the site of stimulation; thus, there are unlikely to be time-dependent changes in the recorded ECoG of ictal events (McNamara, 1986; Scaravilli, 1997; Schubert, et al., 2005; Scharfman, 2008).
3. It generates epileptiform spike-and-wave rhythms which exhibit less variability with regard to their electrographic characteristics than the spontaneous ictal events and interictal discharges produced by other animal models of focal neocortical epilepsies (Kubová & Mares, 1995; Nelson, et al., 2010). This uniformity enables averaging of ictal spike-associated impedance changes to improve the signal-to-noise ratio (SNR).
4. Neocortical focal epilepsies are among the most difficult to treat surgically whilst avoiding postoperative neurological deficit, particularly if the ictal onset zone is localised to eloquent cortex or in the surrounding cortical tissue (Rosenow & Lüders, 2001). Thus, the cerebral cortex was an appropriate target to establish a seizure focus to test the technical accuracy of EIT to image fast neural impedance changes associated with ictal discharges.
   1. **Functional mapping of the primary somatosensory cortex**

The positions of the two stimulating electrodes above the sensorimotor cortex for inducing seizures were decided by mapping the forelimb and hindlimb representations of the primary somatosensory cortex. To do this, SEPs were induced by electrically stimulating peripheral nerves (median, ulnar and radial) in the contralateral forelimb and hindlimb with 1 mA pulses at 2 Hz (pulse width: 500 µs), delivered by a NeuroLog current stimulus isolator (NL800A; Digitimer Ltd, Welwyn Garden City, UK). The SEPs were recorded from the somatosensory cortex with the epicortical electrode array and averaged for 30 s. The ECoG channels exhibiting the highest-amplitude SEPs were considered to be positioned at the centre of the forelimb or hindlimb representation, and the sensorimotor area, defined as the boundary between the hindlimb somatosensory and motor cortex (M1/S1HL), was located accordingly.

Across all animals, the forelimb and hindlimb cortical regions were centred on the same electrodes. Therefore, the same two electrodes were used to stimulate the sensorimotor cortex and induce a consistent ECoG pattern of epileptiform activity in all animals. Furthermore, the ictal focus in the ECoG, defined as the trace containing the highest-amplitude ictal spike-and-wave discharges which was used as the trigger channel for spike sorting, was recorded from the same electrode in all rats. Therefore, because the interelectrode distance was consistent in all experiments, it could be assumed that any functional variabilities across rats amounted to ≤0.6 mm, the diameter of one electrode contact on the epicortical array.

- 1. **Detection and classification of ictal discharges for averaging**

Seizures induced by electrical stimulation of the sensorimotor cortex comprised a characteristic pattern of repeatable 2-5 Hz ictal SWDs which typically occurred during the first half of seizures. The SWDs were occasionally followed by other patterns of epileptic discharges, including sharp waves, polyspike-and-slow wave complexes and fast runs of interictal spikes; since these were more variable across recordings and rats, they were excluded from further analysis. Detection and classification of ictal spikes was performed using an automated spike classification algorithm to isolate SWDs from each seizure epoch based on several distinctive spike features, including shape, amplitude and interspike interval duration (Quiroga, et al., 2004). The algorithm: (a) detects all ictal spikes using a manually set amplitude threshold of 1 mV; (b) calculates the wavelet transform for each detected spike and selects the optimal coefficients for separating spike classes; and (c) uses these wavelet coefficients to perform superparamagnetic clustering of spikes (Quiroga, et al., 2004). The algorithm generates an array of trigger marker timings corresponding to the peak amplitudes of all detected spikes in each cluster, which were manually inspected and altered where necessary. These trigger markers were then used for averaging SWD-related impedance changes. All spike waveforms other than SWDs were excluded from further analysis.

1. *Supplementary Results*
   1. **Spatial arrangement of ECoG and impedance recordings**


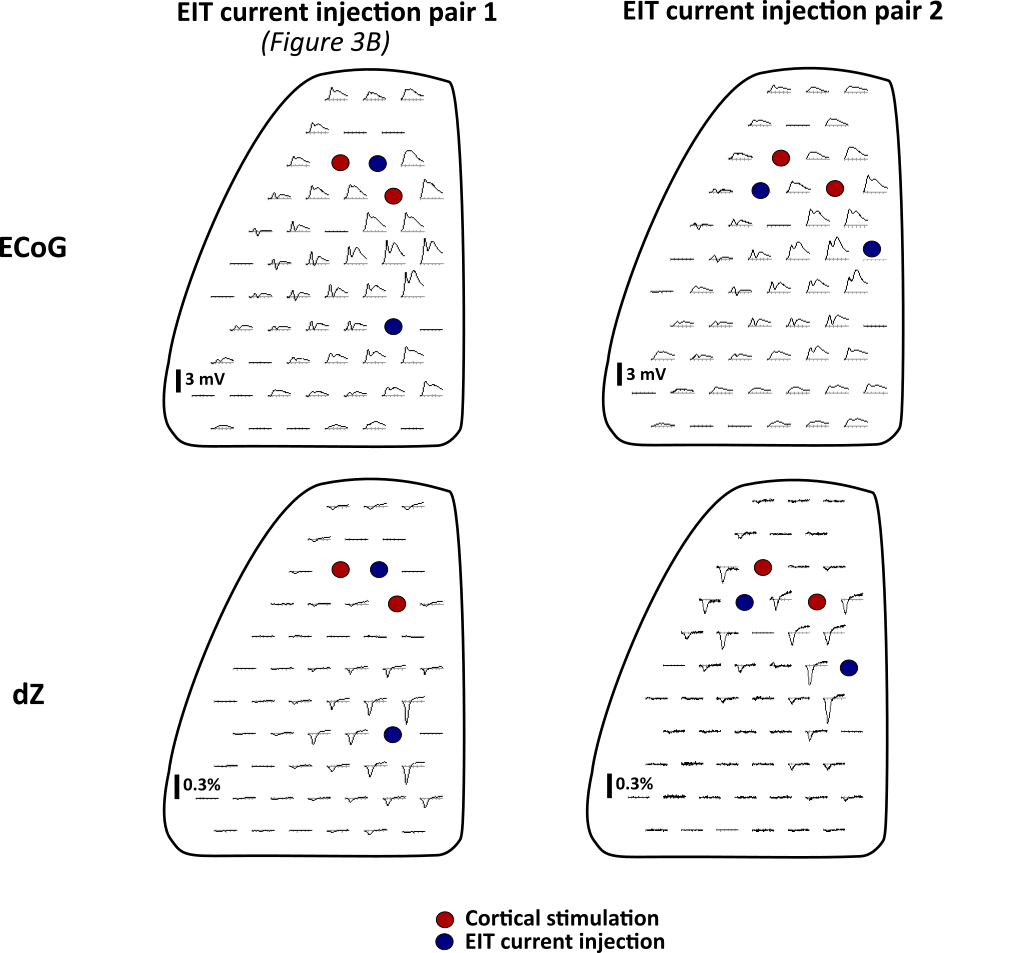


**Supplementary Figure S2. Comparison of ECoG and impedance (dZ) recordings obtained from two different EIT current injecting electrode pairs.** The highest-amplitude dZ traces were consistently recorded in the vicinity of the current injecting points.

***References***

Fisher, R. S. (1989). Animal models of the epilepsies. *Brain Res Rev, 14*(3), 245-278.

Haugvicová, R., Bílková, E., Kubová, H., & Mares, P. (2002). Effects of classical antiepileptics on thresholds for phenomena induced by cortical stimulation in rats. *J Pharm Pharmacol, 54*(7), 1011-1015.

Koronfel, M. A., Aristovich, K., Vongerichten, A. N., & Holder, D. S. (2014). 120-channel electrode arrays for rat brain: towards 3D imaging. Ganonoque, Canada: 15th Int Conf on Biomedical Applications of Electrical Impedance Tomography.

Kubová, H., Lanstiakova, M., Mockova, M., Mares, P., & Vorlicek, J. (1996). Pharmacology of cortical epileptic afterdischarges in rats. *Epilepsia, 37*(4), 336-341.

Kubová, H., & Mares, P. (1995). Suppression of cortical epileptic afterdischarges by ketamine is not stable during ontogenesis in rats. *Pharmacol Biochem Behav, 52*(3), 489-492.

McNamara, J. A. (1986). Kindling model of epilepsy. *Adv Neurol, 44*, 303-318.

Nelson, T. S., Suhr, C. L., Lai, A., Halliday, A. J., Freestone, D. R., McLean, K. J., . . . Cook, M. J. (2010). Seizure severity and duration in the cortical stimulation model of experimental epilepsy in rats: a longitudinal study. *Epilepsy Res, 89*(2-3), 261-270.

Quiroga, R. Q., Nadasdy, Z., & Ben-Shaul, Y. (2004). Unsupervised spike detection and sorting with wavelets and superparamagnetic clustering. *Neural Commut, 16*(8), 1661-1687.

Rosenow, F., & Lüders, H. (2001). Presurgical evaluation of epilepsy. *Brain, 124*(Pt 9), 1683-1700.

Scaravilli, F. (1997). *Neuropathology of epilepsy.* New Jersey: World Scientific .

Scharfman, H. E. (2008). Epilepsy as an example of neural plasticity. *Neuroscientist, 8*(2), 154-173.

Schubert, M., Siegmund, H., Pape, H., & Albrecht, D. (2005). Kindling-induced changes in plasticity of the rat amygdala and hippocampus. *Learn Mem, 12*(5), 520-526.
